# Nitroprusside modulates pulmonary vein arrhythmogenic activity

**DOI:** 10.1186/1423-0127-17-20

**Published:** 2010-03-20

**Authors:** Yung-Kuo Lin, Yen-Yu Lu, Yao-Chang Chen, Yi-Jen Chen, Shih-Ann Chen

**Affiliations:** 1 Taipei Medical University, Graduate Institute of Clinical Medicine, Taipei, Taiwan; 2 Division of Cardiovascular Medicine, Taipei Medical University, Wan Fang Hospital, Taipei, Taiwan; 3 Department of Biomedical Engineering, National Defense Medical Center, Taipei, Taiwan; 4 National Yang-Ming University, School of Medicine, Division of Cardiology and Cardiovascular Research Center, Veterans General Hospital, Taipei, Taiwan; 5 Division of Cardiology, Sijhih Cathay General Hospital, Sijhih, Taiwan

## Abstract

**Background:**

Pulmonary veins (PVs) are the most important sources of ectopic beats with the initiation of paroxysmal atrial fibrillation, or the foci of ectopic atrial tachycardia and focal atrial fibrillation. Elimination of nitric oxide (NO) enhances cardiac triggered activity, and NO can decrease PV arrhythmogensis through mechano-electrical feedback. However, it is not clear whether NO may have direct electrophysiological effects on PV cardiomyocytes. This study is aimed to study the effects of nitroprusside (NO donor), on the ionic currents and arrhythmogenic activity of single cardiomyocytes from the PVs.

**Methods:**

Single PV cardiomyocytes were isolated from the canine PVs. The action potential and ionic currents were investigated in isolated single canine PV cardiomyocytes before and after sodium nitroprusside (80 μM,) using the whole-cell patch clamp technique.

**Results:**

Nitroprusside decreased PV cardiomyocytes spontaneous beating rates from 1.7 ± 0.3 Hz to 0.5 ± 0.4 Hz in 9 cells (P < 0.05); suppressed delayed afterdepolarization in 4 (80%) of 5 PV cardiomyocytes. Nitroprusside inhibited L-type calcium currents, transient outward currents and transient inward current, but increased delayed rectified potassium currents.

**Conclusion:**

Nitroprusside regulates the electrical activity of PV cardiomyocytes, which suggests that NO may play a role in PV arrhythmogenesis.

## Background

Atrial fibrillation (AF) is the most common sustained arrhythmia in clinical medicine. The pulmonary veins (PVs) have been demonstrated to be an important source of the initiation of AF [1,2] and also to have a role in the maintenance of AF [3]. Previous anatomical and electrophysiological study in isolated PVs specimen have demonstrated that PVs contain a mixture of pacemaker cells and working myocardium [4-9]. In canine PVs, we also demonstrated that PVs have arrhythmogenic activity through the enhancement of spontaneous activities or high frequency irregular rhythms [10,11]. These findings confirmed the previous observation in embryological heart, whereas PVs were suggested to work as a subsidiary pacemaker [12]. Enhancement of automaticity and triggered activity in PV cardiomyocytes with pacemaker activity was suggested to play a critical role in the pathophysiology of AF [10,11,13,14].

Previous studies have shown that nitric oxide (NO) has important regulatory effects on the cardiovascular system [15,16]. NO has been shown to have a role in the development of triggered arrhythmias generated by Ca^2+^overload [17]. Our previous study in vivo also showed that NO could suppress trigged activity induced ventricular tachycardia [18]. It is known that PVs contain endothelium and smooth muscle which may produce NO through the enzyme of eNOS or iNOS. In addition, cardiac myocytes also express eNOS activity [19]. NO has been shown to regulate PV arrhythmogensis through mechano-electrical feedback [20]. Because PVs was known to induce atrial arrhythmia through the enhancement of triggered activity, it is possible that NO may play a critical role in the PV arrhythmogenic activity. Moreover, perioperative administration of nitroprusside (NO donor) during the rewarming period could prevent postoperative AF in patients undergoing myocardial revascularization, which suggests the anti-AF effects of nitroprusside [21]. However, it is not clear whether nitroprusside may have direct electrophysiological effects on PV cardiomyocytes. Therefore, the purpose of this study was to study the effects of nitroprusside on the ionic currents and arrhythmogenic activity of single PV cardiomyocytes.

## Materials and methods

### Isolation of single cardiomyocytes

The investigation conformed to the institutional Guide for the Care and Use of Laboratory Animals. Twenty-one mongrel dogs were used in this study. After the dogs were anesthetized with sodium pentobarbital (30 mg/kg, i.v.), the hearts were rapidly removed through a thoracotomy and dissected at room temperature in normal Tyrode solution with the composition (in mM) of 137 NaCl; 4 KCl; 15 NaHCO_3_; 0.5 NaH_2_PO_4_; 0.5 MgCl_2_; 2.7 CaCl_2_, and 11 dextrose. Tyrode solution was equilibrated with a gas mixture of 97% O_2 _-3% CO_2_, with a pH of around 7.4.

For dissection of the PVs, the left atrium was opened by an incision extending from the coronary sinus. The PVs were separated from the left atrium about 5 mm proximal to the junction between PVs and left atrium. The veins were separated from the lung parenchyma through the incisions about 20 mm distal to the ending of myocardial sleeve. The isolated PVs were perfused from the distal end with inside out of PVs through a polyethylene tubing. The other end of the polyethylene tubing was connected to a perfusion pump with a perfusion rate of 500 ml/hr. The proximal end and side branches of PVs were ligated with silk. The PVs were perfusated initially with oxygenated normal Tyrode solution and replaced with Ca^2+^-free Tyrode solution. The perfusate was replaced with oxygenated Ca^2+^-free Tyrode solution containing 3 units/ml collagenase (Sigma Type I) and 0.5 units/ml protease (Sigma, Type XIV). After softening of the PVs, the PVs were cut into fine pieces and gently shaken in 5-10 ml of Ca^2+^-free oxygenated Tyrode solution until single cardiomyocytes were obtained. The solution was then gradually changed to normal oxygenated Tyrode solution. Only cells showing clear cross striations were used. Experiments were carried out within the room temperature (34-36°C). The cells were allowed to stabilize in the bath for at least 30 min before experiments.

### Electrophysiological and pharmacological study

Whole-cell patch-clamp was performed in cardiomyocytes by means of an Axopatch 1D amplifier (Axon Instruments, Calif, USA) at 35 ± 1°C. Borosilicate glass electrodes (o.d., 1.8 mm) were used, with tip resistances of 3-5 MΩ. Before formation of the membrane-pipette seal, tip potentials were zeroed in Tyrode solution. Junction potentials (9 mV) were corrected for action potentials (APs) recording. AP and transient inward currents were measured during superfusion with normal Tyrode solution, pipette solution contained (in mM): KCl 20, K aspartate 110, MgCl_2 _1, Mg_2_ATP 5, HEPES 10, EGTA 0.5, and LiGTP 0.1, Na_2_phosphocreatine 5, adjusted to pH 7.2 with 1 N KOH. The APs were recorded in current-clamp mode and ionic currents in voltage-clamp mode as described previously [11]. A small hyperpolarizing step from a holding potential of -50 mV to a testing potential of -55 mV for 80 ms was delivered at the beginning of each experiment. The area under the capacitative currents was divided by the applied voltage step to obtain the total cell capacitance of 36 ± 3 pF in 43 PV cardiomyocytes. Normally, 60% to 80% series resistance (R_s_) was electronically compensated. AP measurements were begun at 5 minutes after cell rupture. The 50% (APD_50_) and 90% (APD_90_) of the AP duration were measured during 1 Hz electrical stimulation in the PV cardiomyocytes. Micropipettes were filled with a solution containing (in mM) CsCl 130, MgCl_2 _1, Mg_2_ATP 5, HEPES 10, EGTA 10, NaGTP 0.1, and Na_2 _phosphocreatine 5, titrated to a pH of 7.2 with CsOH for the experiments on the L-type calcium current (I_Ca-L_). The micropipettes were filled with a solution containing (in mM) KCl 20, K aspartate 110, MgCl_2 _1, Mg_2_ATP 5, HEPES 10, EGTA 0.5, LiGTP 0.1, and Na_2 _phosphocreatine 5, titrated to a pH of 7.2 with KOH for the experiments on the APs, potassium currents, and transient inward currents. Voltage command pulses were generated by a 12-bit digital-to-analog converter controlled by pCLAMP software (Axon Instruments). Recordings were low pass-filtered at half the sampling frequency.

The I_Ca-L _was measured as an inward current during depolarization from a holding potential of -50 mV to testing potentials ranging from -40 to +60 mV in 10-mV steps for 300 ms at a frequency of 0.1 Hz. The NaCl and KCl in the external solution were replaced by tetraethylammonium chloride and CsCl, respectively.

The transient outward current (I_to_) was studied with a double-pulse protocol. A 30-ms pre-pulse from -80 to -40 mV was used to inactivate the sodium channels, followed by a 300-ms test pulse to +60 mV in 10-mV steps at a frequency of 0.1 Hz. CdCl_2 _(200 μM) was added to the bath solution to inhibit the I_Ca-L_. The I_to _was measured as the difference between the peak outward current and steady-state current. The sustained outward potassium currents (I_Ksus_) were measured as the outward current density at the end of the steady state.

The delayed rectified outward potassium current (I_K_) was measured from the peak outward current at the end of 1 s and the depolarization from -40 to +60 mV in 10-mV steps at a frequency of 0.1 Hz during the infusion of CdCl_2 _(200 μM) and 4-aminopyridine (2 mM) in the bath solution.

Transient inward current was induced at clamped potentials from -40 to +40 mV for the duration of 2 sec and then repolarized to -40 mV. The amplitude of transient inward current was measured as difference between the peak of the transient current and the mean of current just before and after the transient current.

### Statistics

All quantitative data are expressed as mean ± SE. The differences between before and after drugs administration were analyzed by Wilcoxon signed-rank test. A P value lower than 0.05 was considered to be statistically significant.

## Results

### Effects of nitroprusside on arrhythmogenic activity of PV cardiomyocytes

**Figure 1:**
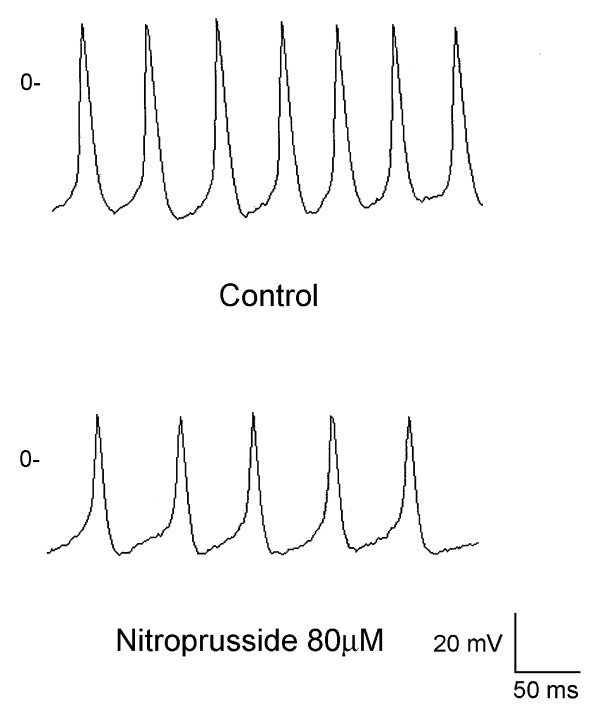
**Spontaneous activity before (upper panel) and after (lower panel) the administration of nitroprusside**. Nitroprusside suppressed the spontaneous activity in a pulmonary vein (PV) cardiomyocyte.

**Figure 2:**
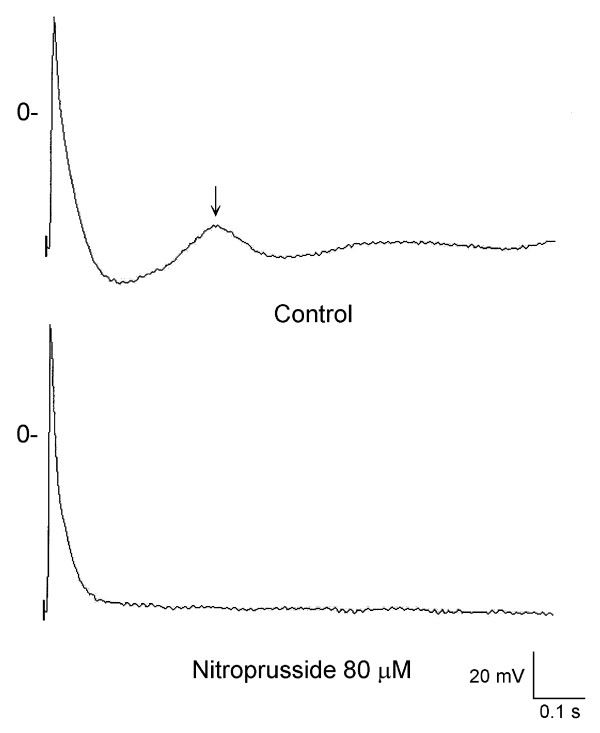
**Effect of nitroprusside on the PV delayed after depolarization**. Delayed afterdepolarization (indicated by arrow) in a PV cardiomyocyte was suppressed after (lower panel) the administration of nitroprusside.

Nine PV cardiomyocytes with pacemaker activity received the administration of 80 μM nitroprusside. Nitroprusside decreased PV cardiomyocytes spontaneous beating rates from 1.7 ± 0.3 Hz to 0.5 ± 0.4 Hz in 9 cells (P < 0.05). Figure 1 shows the example that PV cardiomyocytes spontaneous activity was suppressed from to after the administration of nitroprusside. In addition, nitroprusside inhibited delayed afterdepolarization in 4 (80%) of 5 PV cardiomyocytes. The amplitude of delayed afterdepolarization was suppressed from 9 ± 2 to 4 ± 2 mV. Figure 2 shows the example of the changes of delayed afterdepolarization after nitroprusside.

**Figure 3:**
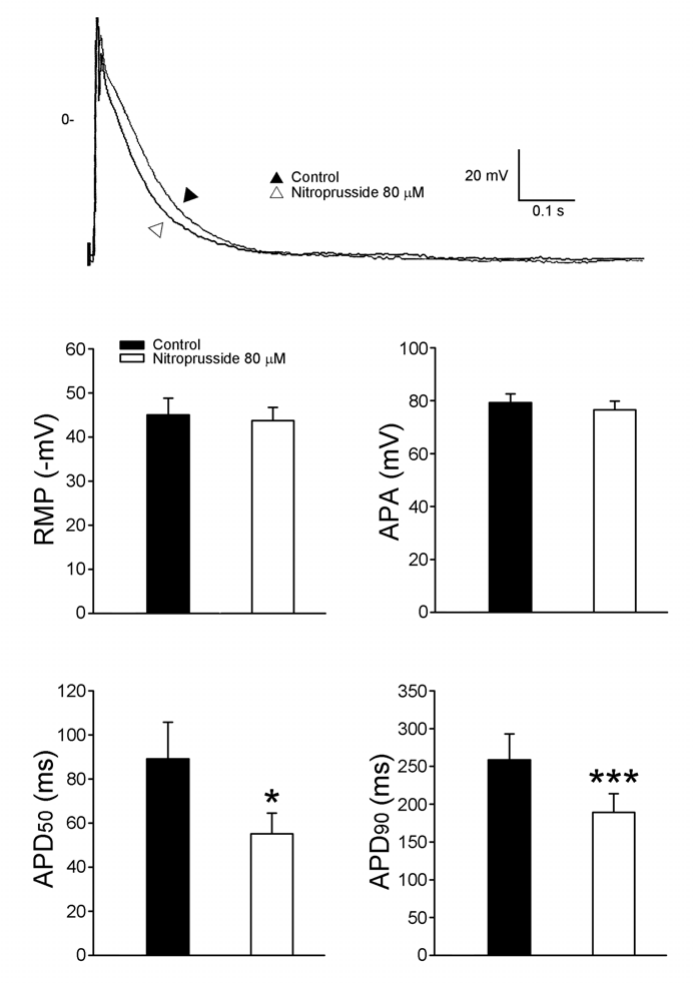
**Effect of nitroprusside on the PV electrical activity**. Action potential of PV cardiomyocyte before (indicated by black triangle) and after (indicated by blank triangle) the administration of nitroprusside. There were shorter action potentials after the administration of nitroprusside. *P < 0.05, versus before nitroprusside PV cardiomyocytes. (RMP: resting membrane potential, APA: action potential amplitude, APD_50_: 50% of action potential duration, APD_90_: 90% of action potential duration).

Figure 3 shows the effects of nitroprusside on AP morphology in the PV cardiomyocytes, nitroprusside (80 μM) shortened the 90% and 50% of the AP duration. Figure 1 shows the example of AP before and after the administration of nitroprusside. However, nitroprusside did not change the resting membrane potential in these cardiomyocytes.

### Effects of nitroprusside on ionic currents of PV cardiomyocytes

**Figure 4:**
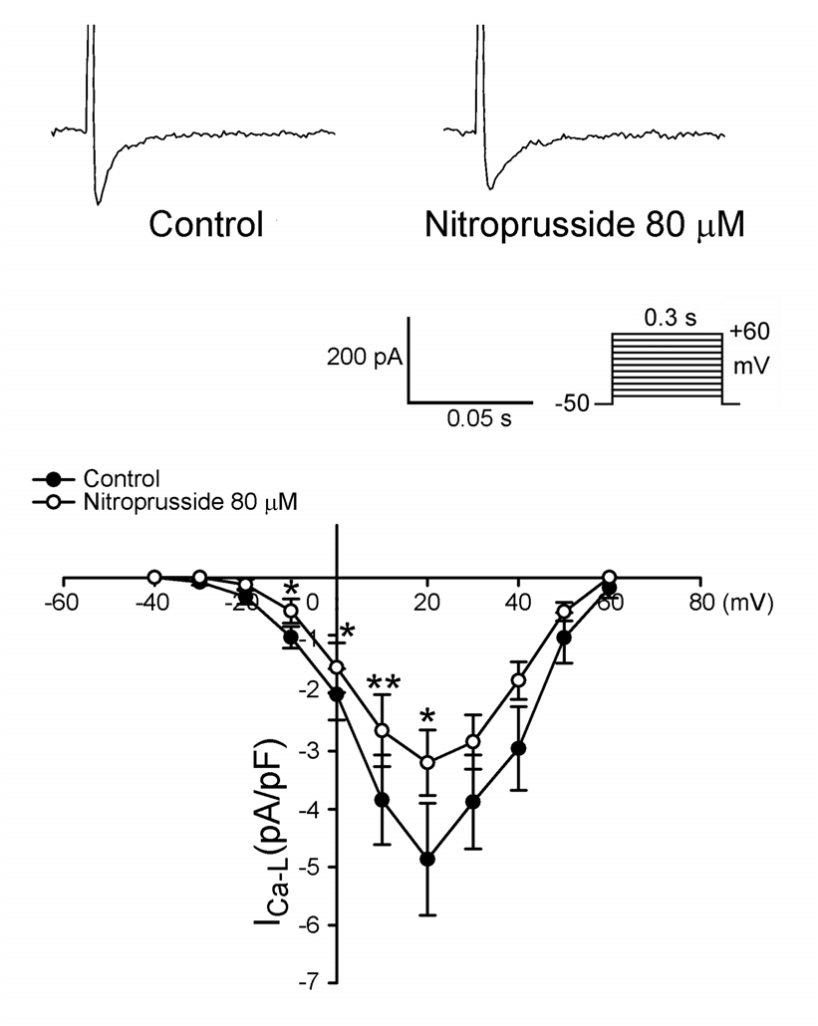
**The current tracings and I-V relationship of the L-type calcium current (I_Ca-L_) of the control and nitroprusside-treated PV cardiomyocytes (n = 6)**. The I_Ca-L _in the nitroprusside-treated PV cardiomyocytes was smaller than that in the control PV cardiomyocytes. The insets of the current tracings show the various clamp protocols. *P < 0.05, **P < 0.01, versus before nitroprusside PV cardiomyocytes.

**Figure 5:**
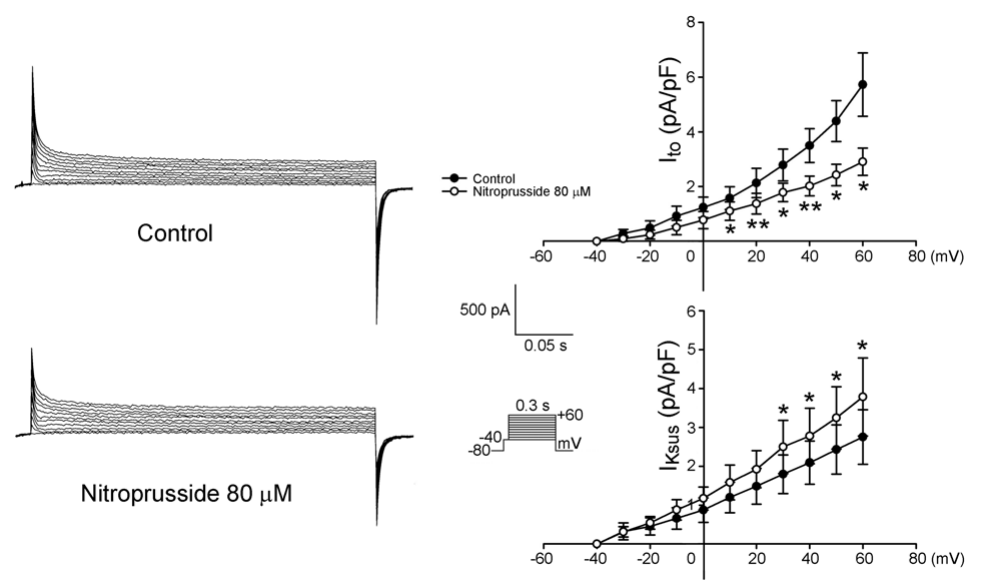
**Effects of nitroprusside on the transient outward current (I_to_) and sustained outward potassium currents (I_Ksus_) in the PV cardiomyocytes (n = 6)**. Upper and lower panel shows the current tracings and I-V relationship of the I_to _of before and after the administration of nitroprusside in PV cardiomyocytes. The I_to _in the nitroprusside-treated PV cardiomyocytes was smaller than that in the control PV cardiomyocytes. Lower panel shows the current tracings and I-V relationship of the I_Ksus _before and after the administration of nitroprusside in PV cardiomyocytes. The I_Ksus _was larger in the nitroprusside-treated PV cardiomyocytes than that in the control PV cardiomyocytes. The insets of the current traces show the various clamp protocols. *P < 0.05, **P < 0.01, versus before nitroprusside PV cardiomyocytes.

**Figure 6:**
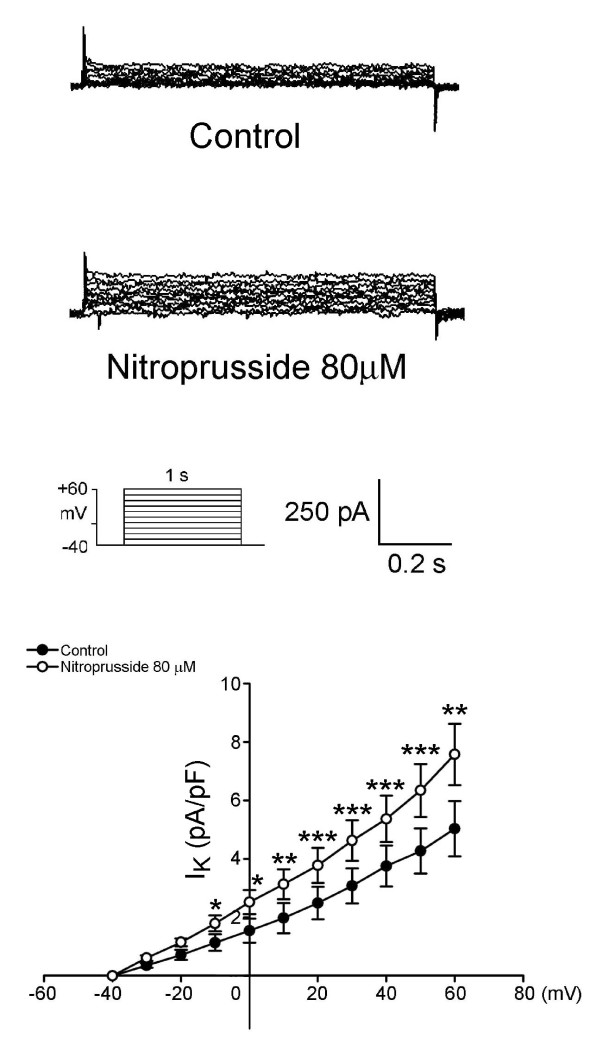
**Effect of nitroprusside on delayed rectified outward potassium current (I_K_) in the PV cardiomyocytes (n = 11)**. Current traces (upper panel) and I-V relationship (lower panel) of I_K _in the PV cardiomyocytes. Insets show the various clamp protocols. *P < 0.05, **P < 0.01, ***P < 0.005, versus before nitroprusside PV cardiomyocytes.

**Figure 7:**
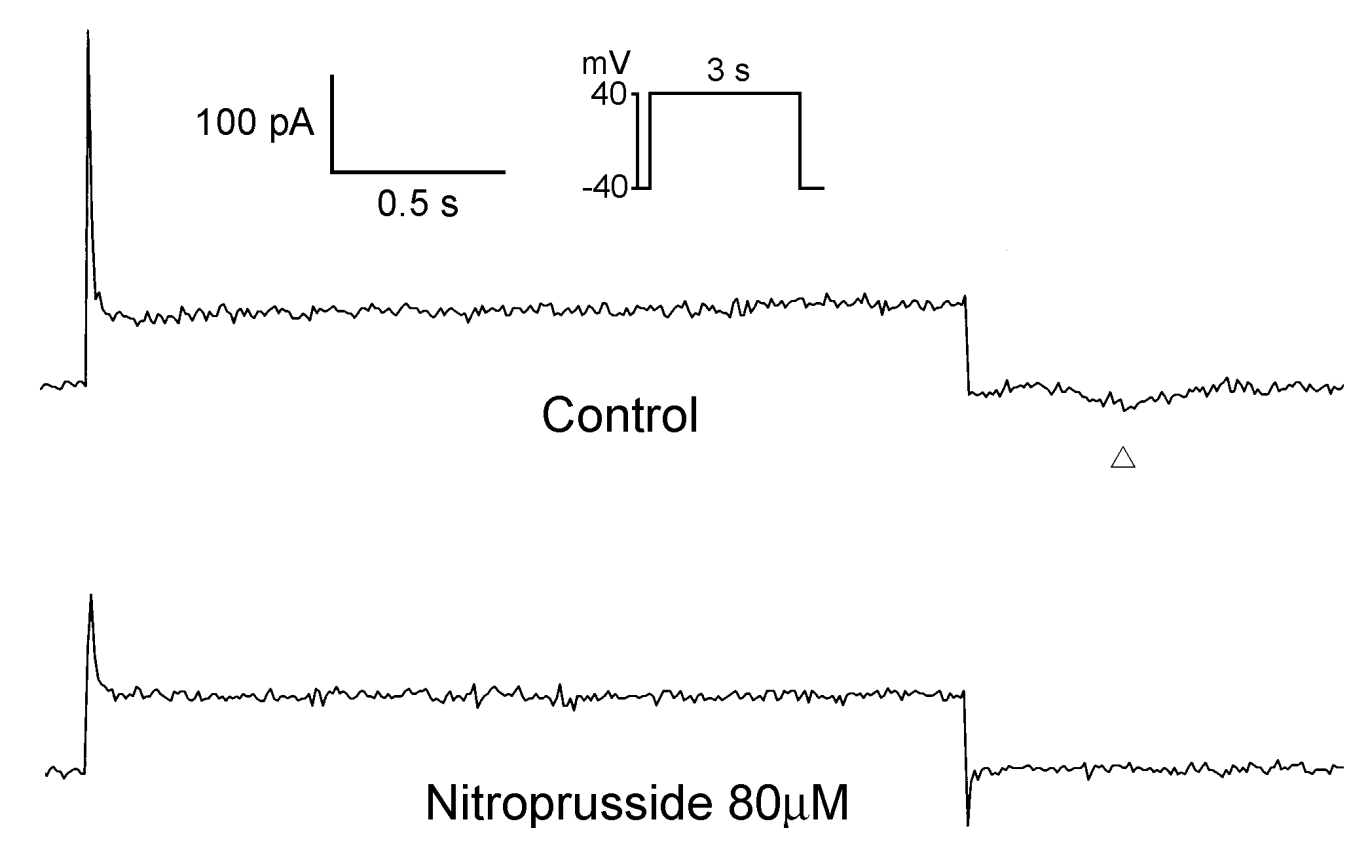
**Effect of nitroprusside on transient inward current (I_ti_) in PV cardiomyocytes**. Current traces of transient inward current indicated by blank triangle before (upper panel) and after (lower panel) the administration of nitroprusside in a PV cardiomyocyte. There was decreased transient inward current after nitroprusside. The transient inward current was induced from repolarization to -40 mV after a depolarizing pulse (from -40 to +40 mV for 3 s, see inset for clamp protocol).

As the example shown in Figure 4, nitroprusside can decrease the I_Ca-L _in PV cardiomyocytes. Moreover, nitroprusside can decrease the I_to _in PV cardiomyocytes, but increase the I_Ksus _(Figure 5). Figure 6 shows the current traces and I-V relationship of I_K _in PV cardiomyocytes before and after the administration of nitroprusside. Nitroprusside enhanced I_K _in PV cardiomyocytes. Figure 7 shows the tracing of transient inward current before and after the administration of nitroprusside, whereas nitroprusside inhibited transient inward current from 0.74 ± 0.13 pA/pF to 0.29 ± 0.09 pA/pF (n = 13, P < 0.001).

## Discussion

In this study, for the first time, we demonstrated that nitroprusside can directly suppress the spontaneous activity, inhibit delayed afterdepolarization with the decreases of transient inward currents in PV cardiomyocytes. NO has been shown to change automaticity in sinoatrial cell [22] and also been found to have lower plasma NO levels in the patients with AF than subjects with sinus rhythm [23]. These findings suggested that NO may be associated with the occurrence of AF. Previous studies have shown that PVs play a critical role in the genesis of atrial fibrillation. Our studies further showed that PVs have a high arrhythmogenic activity through the enhancement of automaticity and triggered activity [11,13,14]. In this study, we demonstrated that NO could inhibit PV cardiomyocytes spontaneous activity and also suppressed triggered activity from DAD. These findings confirmed that NO has a role in the PV arrhythmogenic activity.

NO have been shown to alter intracellular Ca^2+ ^homeostasis or inhibit both L-type Ca^2+ ^currents and the stimulation of L-type Ca^2+ ^currents by β-adrenergic agonists [24-26]. Action of NOS3 has been shown to produce NO to enhance Ca^2+ ^sensitivity of the slowly activating delayed rectified potassium current with the shortening of AP duration in guinea pig ventricular cardiomyocytes [27]. Similarly, this study found that direct administration of NO donor can decrease I_Ca-L_, which may contribute to the shortening of AP duration and the decreases of PV spontaneous activity and triggered activity. Previous studies have indicated the importance of calcium homeostasis in PV electrical activity [28-30] and transient inward current plays an important role in the genesis of PV arrhythmogenesis [11,13,14]. In this study, we demonstrated that NO suppress transient inward currents, which may result in the suppression of triggered activity in PV cardiomyocytes. I_Kur _has been found in atrial or PV cardiomyocytes. In this study, we measured the I_Ksus _because the current density and I-V relationship of I_Ksus _and I_Kur _are quite similar [31], and found an increase of the I_Ksus _after administration of nitroprusside, which may result in the shortening of AP duration in PV cardiomyocytes. Moreover, nitroprusside increase the I_K _also can shorten the AP duration in PV cardiomyocytes.

### Potential limitations

The data in this study should be interpreted with caution due to the potential limitations. Only single dosage of nitroprusside was used in this study. It is not clear whether nitroprusside has concentration-dependent effects on the PVs. Additionally, although nitroprusside is considered to be NO donor, without the use of NO scavenger to reduce or reverse the effects of nitroprusside, we may not exclude the possibility that our findings may not be NO relevant. Moreover, NO signaling in cardiac muscle was reported to be caused by the stimulation of guanylate cyclase/cGMP production or nitrosylation of sulfhydryl groups on cystein residue of channel protein [32,33]. However, this study did not investigate the molecular mechanisms underlying the ionic effects of nitroprusside on PV cardiomyocytes.

## Conclusions

Nitroprusside has significant effects on regulating the arrhythmogenic activity of PV cardiomyocytes, which suggests that NO may play a role in PV arrhythmogenesis.

## Competing interests

The authors declare that they have no competing interests.

## Authors' contributions

YKL and YYL interpreted the data and drafted the manuscript. YCC performed the experiments and revised it for scientific content. YJC and SAC conceived of this study, and participated in its design and coordination. All authors read and approved the final manuscript.
